# How I do it: microvascular decompression for hemifacial spasm using an inferior ventrolateral brainstem approach

**DOI:** 10.1007/s00701-026-06977-2

**Published:** 2026-07-07

**Authors:** Gustavo Polo, Joseph Maarrawi, Marc Sindou

**Affiliations:** 1 Department of Neurosurgery, Hôpital Neurologique Pierre Wertheimer, 59 Boulevard Pinel, 69677 Bron Cedex, Lyon, France; 2 Department of Neurosurgery, Faculty of Medicine, University Medical Centre Hotel-Dieu de France, Hospital and Laboratory of Research in Neuroscience, PTS, Saint Joseph University, Beirut, Lebanon; 3 University of Lyon, France; Clinique Bretéché Groupe ELSAN, Nantes, France

**Keywords:** Facial nerve, Hemifacial spasm, Intra-operative neuromonitoring, Microvascular decompression, Neurovascular conflict, Vascular compression syndrome

## Abstract

Hemifacial spasm is commonly caused by a vascular compression of the facial nerve at its root exit zone (REZ), at brainstem. Microvascular decompression provides long-term cure in approximately 90% of the patients (delayed in one-third). Based on more than three hundred procedures, the authors advocate an inferior ventro-lateral brainstem approach. This trajectory optimizes access to the REZ, while minimizing traction on the cerebellum, the cochleo-vestibular nerve, and the labyrinthine artery. Decompression should be achieved by transposing the offending vessel (PICA, AICA, either alone or associated with a vertebro-basilar- dolichoectatic artery). Monitoring of brainstem auditory evoked potentials can be useful during learning curve.

**Supplementary Information:**

The online version contains supplementary material available at https://doi.org/10.1007/s00701-026-06977-2.

## Introduction

**Fig. 1:**
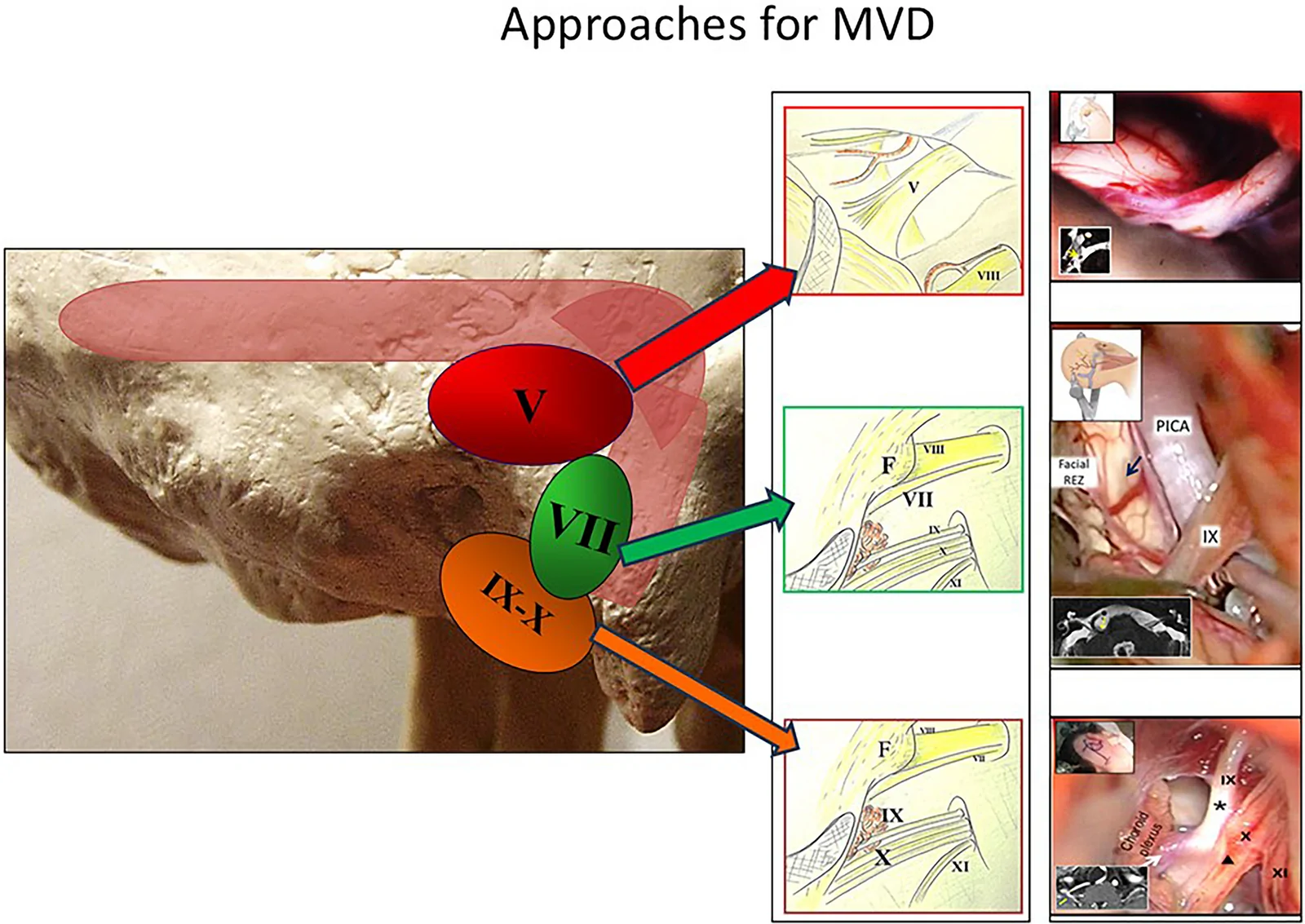
Recommended locations for key-hole craniectomies to access CNs in the cerebello-pontine-angle (CPA) for respective Microsurgical Vascular Decompression (MVD)**,** established in 1992 from intra-operative BAEPs recordings [9]. Left: Schematic representation of the recommended craniectomies for approaching the CPA CNs and for minimize risk of hearing impairment. Middle (schematic drawings) and right (microsurgical views) columns illustrating (from top to bottom), the following approaches: Infratentorial-supracerebellar to access CN V for Trigeminal neuralgia, Infrafloccular to access CN VII for Hemifacial spasm, Retrocondylar to access CN IX-X for Vago-glossopharyngeal neuralgia

Hemifacial spasm -a disabling condition- can be managed by two different treatment modalities, a palliative: botulinum toxin injections, a curative: the microsurgical vascular decompression (MVD) [1]. Considering that the far-most frequent site of culprit neuro-vascular conflicts (NVCs) is the facial-Rez (Root Exit Zone), an inferior approach to directly access the ventro-lateral aspect of the brainstem is advocated [9]. Further, coming from below via an infrafloccular trajectory [6] avoids stretching the cochlea-vestibular cranial nerve (CN) complex, which may be responsible for hearing and/or gait disturbances [9]. Based on more than three hundred procedures the present article aims to justify and describe the inferior ventro-lateral approach to brainstem (Fig. 1).

## Relevant surgical anatomy

### Facial nerve

Although the offending vessel can be located along any intradural segment of the facial nerve most of the NVCs occur at REZ at brainstem, where the nerve fibers are covered by central myelin (CM), oligodendrocyte-derived. To be recalled, the facial REZ corresponds to the point where the nerve emerges from brainstem in the medullary-pontine sulcus. The CM portion of the root—3 to 5 mm in length [3]—extends from REZ to the Transitional Zone where it joins the peripheral segment**,** characterized by Schwann-cell myelin.

The NVCs -classically described at level of the REZ- may actually occur more proximally along the facial fibers, in their (2–8 mm) subpial course [2, 4] or even more deeply in their intra-axial trajectory.

Ectopic excitation and/or ephaptic transmission at the site of nerve injury may induce antidromically—a synaptic reorganization in the facial nucleus, resulting in neuronal hyperactivity. This hyperactivity is clinically reflected by spontaneous contractions and synkinetic spasms of the facial muscles, as well as by the lateral spread muscle responses.

### Cochleo-vestibular nerve complex

The VIIIth nerve **is** particularly at risk as it is encountered early in the operative field, whereas the facial REZ lies more ventrally. The VIII CN vulnerability is related to its relatively long (12 mm) cisternal CM portion.

### Vessels

The offending vessels are the posterior-inferior cerebellar artery (PICA) and the anterior-inferior cerebellar artery (AICA), alone or with a dolichoectatic vertebrobasilar artery (in combination in 20%, alone in 4%).

Access to these arteries follows the «Rule of Three” concept developed by Rhoton and Matsushima for navigation in the CPA [5].

Particular attention must be paid to preserving the arterial perforators responsible for brainstem supply. Also, the AICA and its tributary: the labyrinthine artery, which supply cochlea, must be carefully protected.

## Operative technique

### Pre-operative assessment

Pure tone audiogram and, when available, brainstem auditory evoked potentials are essential to detect eventual pre-existing auditory abnormalities, as well as they provide a baseline for comparison in the event of post-operative disturbances.

High-resolution MRI **-**with the three following sequences: 3D-T2 (for nerves), 3D -TOF- Angio (for arteries), and 3D-T1 + Gadolinium (additionally for veins) **-**is crucial for surgical planning.

Additionally on TOF-Angio-MRI, it is verified that there is no VBA posterior procidence at the occipito-atlas junction, which would make VBA at risk on deep muscular dissection.

### Patient positioning

The patient, under general anesthesia with endotracheal intubation, is positioned in lateral (park-bench) position, with contralateral neck flexion to avoid obstruction by shoulder. Head (slightly flexed and rotated 15° toward contralateral side) is moderately elevated to facilitate venous return. Traction on shoulder should not be excessive, to avoid stretching of the brachial plexus.

### Surgical approach

**Fig. 2:**
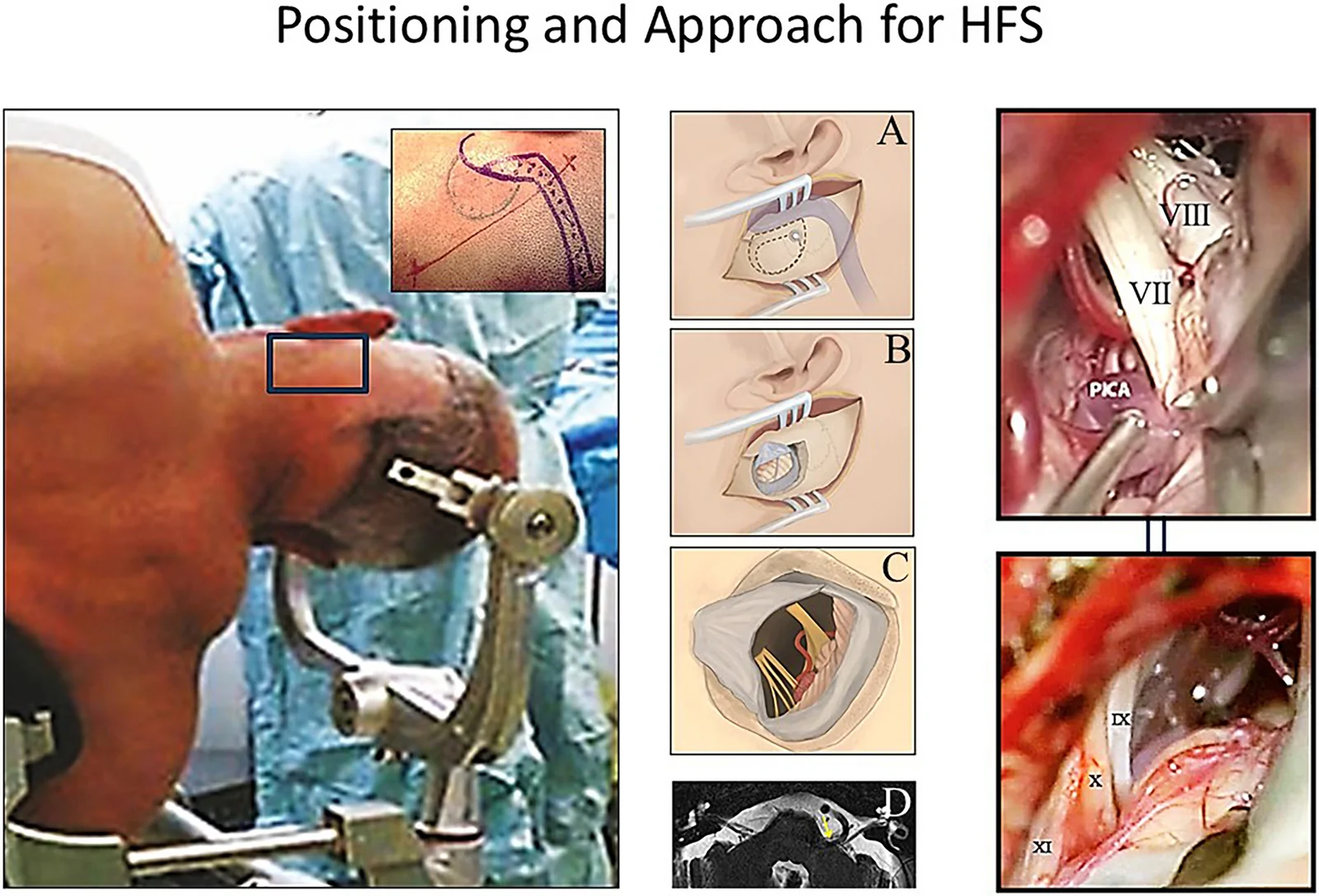
Positioning and approach. Left: Patient positioning in lateral decubitus position. Middle (from top to bottom): steps of the retromastoid-retrosigmoid approach for MVD of the facial nerve. Right inferior image: intradural approach started from below, showing the XI, X, IX CNs; Right superior image: upwards, the PICA loop is separated from brainstem, allowing view of facial nerve ventrally to the VIIIth

For targeting the VIIth REZ from below, the surgical approach is designed so that the keyhole craniectomy is centered posterior to the tip of the mastoid process (Fig. 2). Importantly to be mentioned, for targeting the Vth CN, the keyhole would be centered adjacent and inferior to the transverse sinus so that the trajectory be infratentorial-supracerebellar.

### Skin incision, craniectomy, and dural opening

A skin incision of at least 5 cm is followed by a low lateral retromastoid craniectomy (~ 2 × 1.5 cm), exposing the posterior edge of the sigmoid sinus. Craniectomy starts with drilling a burr-hole and is enlarged -as needed- by additional bone resection using Kerrison rongeur and/or drilling. If mastoid cells are opened, they are occluded with a piece of fat, and -if necessary- additionally sealed with bone wax.

After dural incision, the dural flap is reflected on the sigmoid sinus to access the CPA cistern from below. A narrow blade of the Sugita-Type, mounted on a self-retaining retractor of the Yasargil-type, is gently placed on the inferolateral aspect of the cerebellum to avoid it falling down into the way. Use of a self- retractor has the advantage to liberate the surgeon's hand from retraction-function, and to help maintaining open the corresponding cerebellar/brainstem fissure(s).

BAEPs monitoring can be initiated at this stage.

### Access to the VIlth REZ at brainstem

Under the operative microscope, the arachnoid is opened from the lower CNs up to the flocculus and the CN VIII. The flocculus is then elevated after detaching its adhesions to the choroid plexus (sometimes hypertrophic) and the VIII CN complex. Noteworthy, trajectory does not encounter major veins.

This trajectory provides direct access to the pontomedullary sulcus, and the inferior aspect of the facial REZ, -at the ventrolateral aspect of brainstem, most frequently the site of the offending vessel(s).

### Identification of the offending vessels

**Fig. 3:**
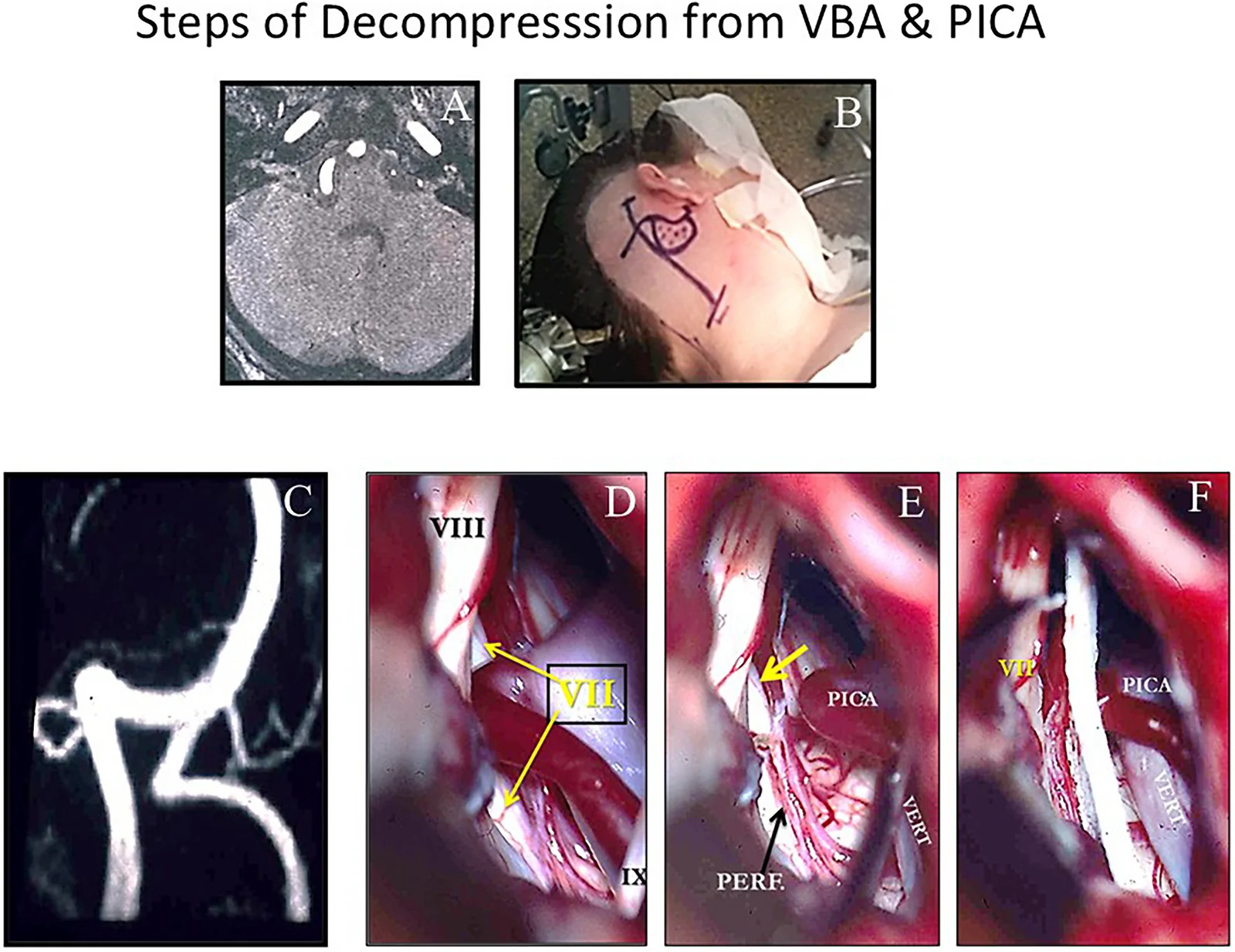
Steps of decompression from Posterior Inferior Cerebellar Artery (PICA) and Vertebro Basilar Artery (VBA). Neurovascular conflict by PICA. **A** Axial MRI showing VBA and dolichoectatic-PICA conflicting on the right side. **B** Landmarks of skin incision and craniectomy. **C** Angio-TOF reconstruction of VBA and PICA. **D** Microsurgical view of the conflict at the facial REZ (ventrally to the VIIIth CN). **E** mobilization of PICA. Note the perforators to brainstem (PERF). **F** PICA and VBA maintained apart by (semirigid) Teflon plate

The conflicting artery(ies) -PICA, AICA, alone or in association with a **dolichoectatic**-vertebrobasilar artery – is (are) identified and carefully mobilized to preserve perforators (Fig. 3).

### Decompression

**Fig. 4:**
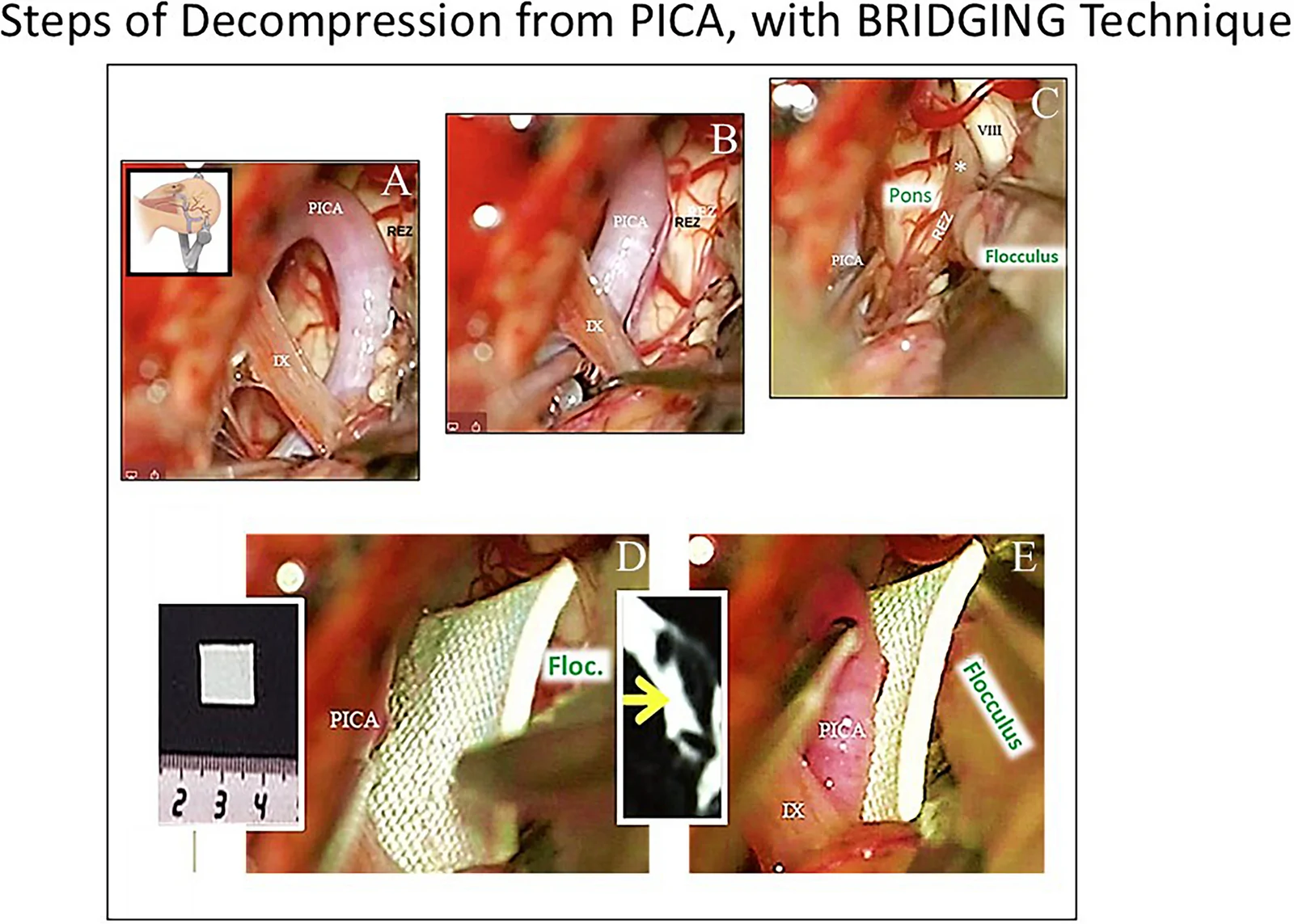
The bridging technique. MVD of a left-sided neurovascular conflict by a dolichoectatic posterior-inferior-cerebellar artery (PICA). Steps of decompression: **A** Approach from below via an inferior-ventro-lateral access to brainstem. Approach starts from lower cranial nerves (see IX nerve). **B** Facial REZ is compressed at brainstem by PICA loop. Note greyish point of the compression, testifying of focal demyelination. **C** After retraction of the flocculus the VIIth nerve exit (asterisk) can be seen ventrally to the VIIIth cranial nerve. **D** Bridging technique by interposition of a semi-rigid knitted Teflon plate inserted between the PICA on one side and the brainstem and flocculus on the other side. **E** Teflon piece in place bridging the facial REZ and the VIIIth nerve complex

Principle is to avoid creating neo-compression of the CN structures. Elongated (still-flexible) arterial loops can be transposed using slings made of shredded Teflon fibers (not shown). Atherosclerotic arteries can be maintained away by interposing a (semi-rigid) knitted piece of Teflon or Dacron, according to the so-called "bridging" technique [8, 11] (Fig. 4).

After decompression, careful inspection ensures that no vessel is kinked and nerves compressed. Vasospasm (if occurring) must be reversed with warm saline irrigation and topical application (of a few) droplets of 10% papaverine solution.

### Closure

Dura is closed with single stitches and, if necessary, an additional patch (from fascia lata). Bone dust may be put back to fill defect though simple closure may be sufficient. Soft tissues are closed in layers, and a compressive dressing applied to reduce risk of pseudo-meningocele.

## Postoperative care

Immediate or delayed CN deficit(s) should be promptly identified and appropriately managed.

No specific medication is required.

Attention is paid to (delayed) CSF rhinorrhea or serous otitis through incontinent mastoid cells.

## Intra-operative neuromonitoring

**Fig. 5:**
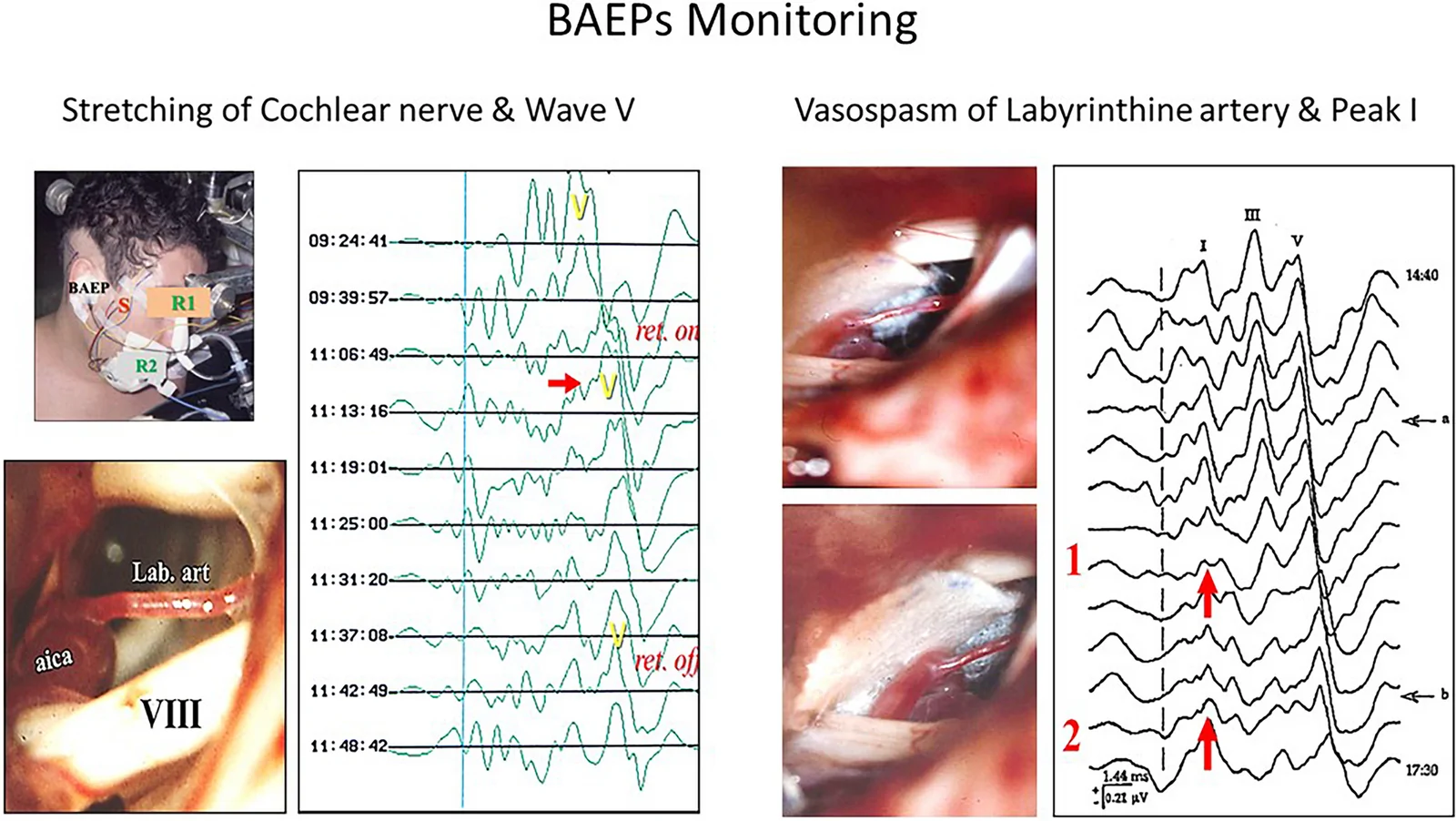
BAEPs monitoring during MVD for right-sided hemifacial spasm [Insert: equipment for BAEP recordings (and facial EMG recordings: not shown)]. Left: Cerebellar retraction causes stretching of CN VIII, resulting in increase of Wave V latency; reversible upon release. Right: Vasospasm of labyrinthine artery leads to decreased peak I amplitude (upper microsurgical view), reversible after droplets of 10% solution papaverine in topical application (lower microsurgical view)

-Brain Auditory Evoked Potentials (BAEPs) assess auditory pathway integrity. An increase of 1 ms in latency of Wave V serves as a warning signal [7]. Abolition correlates with permanent deafness. Decrease in amplitude of Peak I warns integrity of cochlea (Fig. 5).

-EMG-recording of the Lateral spread responses, advocated by Møller, has not proven reliable in predicting completeness of the decompression, at least in in our experience [11].

## How to avoid complications

- Verify positioning so that shoulder does not obstruct access from below to the facial-REZ.
- Craniectomy must be performed with caution because of extreme fragility of the sigmoid sinus wall.
- A too small key-hole craniectomy would entail risk of not-controlling an unexpected event.
- Manipulations of AICA and labyrinthine artery can generate cochlear ischemia. If vasospasm occurs, irrigation with warm saline and droplets of papaverine in 10% solution are recommended.
- Short perforators to brainstem could endanger transposition of the conflicting artery. In such circumstance, a (small) ball of shredded Teflon fibers can be inserted between the culprit vessel and the nerve root.
- Decompression must not result in “neo-compression” of the facial and/or the cochlear-vestibular nerves.
- Watertight closure is crucial to avoid CSF leakage.

## Indication

MVD may be first therapeutic option as medications are ineffective and Botulinum toxin provides only transient benefit. There is no evidence that repeated injections compromise subsequent surgery.

## Patient information

MVD is the only curative treatment, with approximately 90% probability of permanent relief. Resolution of facial spasm may be delayed (to one year in 10%) [10].

Revision should be critically discussed if high-resolution MRI does not evidence a remaining compressive vessel.

Specific complication risks should be detailed in the Informed Consent. In our series, these include: immediate facial palsy (2%, lasting in 1%); secondary—usually transient—facial palsy (3%); permanent hearing loss (1.6%); permanent vertigo and/or tinnitus (1.4%); permanent lower CN deficit (1%) [10]. 

## Conclusion

The Inferior-Ventro-Lateral approach to brainstem, by providing direct access to the facial REZ and compression site, is optimal for minimizing retraction/manipulations of the neighboring vasculo-neural structures.

## 10 key points summaries

1. Primary Hemi-Facial Spasm (HFS) is almost exclusively from vascular compression of arterial origin, which can be preoperatively evidenced using high-resolution MRI.
2. The neurovascular conflict (NVC) is most commonly located in the Facial Root Exit Zone (REZ) at brainstem. It is only rarely a loop in the CPA cistern or inside the internal auditory meatus.
3. The optimal surgical approach is infero-ventro-lateral to the brainstem, using an infra-floccular corridor to the facial REZ, thereby avoiding stretching the cochlear-vestibular nerve complex, in other words: “proceed from below”.
4. Landmark for the (key-hole) craniectomy is posterior to the mastoid tip, allowing entry into the CPA cistern “from below”.
5. Intradural trajectory consists of “climbing” the successive steps of the stairs: the lower cranial nerves (XI, X, IX), then the choroid plexus emerging from the Luschka foramen, so that to reach the facial REZ inferiorly to the flocculus.
6. The most frequent conflicting vessel is PICA, followed by AICA, alone or in combination with its the V-B artery.
7. Decompression should not create neo-compression. In the presence of a dolichoectatic atheromatous/arteriosclerotic artery the so-called bridging technique may be employed.
8. Intraoperative Neuromonitoring (ION), especially BAEP recordings, can be useful, at least before learning curve reaches satisfactory level.
9. Resolution of hemifacial spasm is often delayed (in 30% of our patients, up to one year in 10%). So, decision of redoing surgery should be tempered.
10. Botulinum Toxin injections, frequently administered prior to surgical referral, do not appear to compromise surgical outcome.

## Supplementary Information

Below is the link to the electronic supplementary material.

ESM 1 Supplementary Material 1 (MP4 264 MB)

## Data Availability

No datasets were generated or analysed during the current study.

## References

[1] Barker FG 2nd, Jannetta PJ, Bissonette DJ, Shields PT, Larkins MV, Jho HD (1995) Microvascular decompression for hemifacial spasm. J Neurosurg 82(2):201–210 7815147 10.3171/jns.1995.82.2.0201

[2] Campos-Benitez M, Kaufmann AM (2008) Neurovascular compression findings in hemifacial spasm. J Neurosurg 109:416–420 18759570 10.3171/JNS/2008/109/9/0416

[3] Guclu B, Sindou M, Meyronet D, Streichenberger N, Simon E, Mertens P (2011) Cranial nerve vascular compression syndromes of the trigeminal, facial and vago-glossopharyngeal nerves: comparative anatomical study of the central myelin portion and transitional zone; correlations with incidences of corresponding hyperactive dysfunctional syndromes. Acta Neurochir (Wien) 153:2365–2375 21947457 10.1007/s00701-011-1168-1

[4] Li Y, Zheng X, Hua X, Ying T, Zhong J, Zhang W, Li ST (2013) Surgical treatment of hemifacial spasm with zone-4 offending vessel. Acta Neurochir 155:849–853 23355064 10.1007/s00701-013-1623-2

[5] Matsushima K, Yagmurlu K, Kohno M, Rhoton AL Jr (2016) Anatomy and approaches along the cerebellar-brainstem fissures. J Neurosurg 124:248–263 26274986 10.3171/2015.2.JNS142707

[6] Park HS, Chang DK, Han YM (2009) Infranuchal infrafloccular approach to the more vulnerable segments of the facial nerve in microvascular decompressions for the hemifacialspasm. J Korean Neurosurg Soc 46:340–345 19893723 10.3340/jkns.2009.46.4.340 PMC2773391

[7] Polo G, Fischer C, Sindou M, Marneffe V (2004) Brainstem auditory evoked potential monitoring during microvascular decompression for hemifacial spasm: intraoperative brainstem auditory evoked potential changes and warning values to prevent hearing loss. Prospective study in a consecutive series of 84 patients. Neurosurgery 54:97–106 14683545 10.1227/01.neu.0000097268.90620.07

[8] Rhomberg T, Eordogh M, Lehmann S, Schroeder H (2024) Endoscope-assisted microvascular decompression in hemifacial spasm with a Teflon bridge. Acta Neurochir (Wien) 166(1):239. 10.1007/s00701-024-06142-7 PMC11139744 38814504

[9] Sindou M, Fobé JL, Ciriano D, Fischer C (1992) Hearing prognosis and intraoperative guidance of brainstem auditory evoked potential in microvascular decompression. Laryngoscope 102:678–682 1602916 10.1288/00005537-199206000-00014

[10] Sindou M, Mercier P (2018) Microvascular decompression for hemifacial spasm: Outcome on spasm and complications, a review. Neurochirurgie 64(2)106–116 10.1016/j.neuchi.2018.01.001 29454467

[11] Sindou M, Mercier P (2018) Microvascular decompression for hemifacial spasm: Surgical techniques and intraoperative monitoring. Neurochirurgie 64(2):133–143. 10.1016/j.neuchi.2018.04.003 29784430

